# Immunogenetic characterization of clonal plasma cells in systemic light-chain amyloidosis

**DOI:** 10.1038/s41375-020-0800-6

**Published:** 2020-03-19

**Authors:** Isabel Cuenca, Daniel Alameda, Beatriz Sanchez-Vega, David Gomez-Sanchez, Diego Alignani, Marta Lasa, Esther Onecha, Ramon Lecumberri, Felipe Prosper, Enrique M. Ocio, Maria Esther González, Alfonso García de Coca, Javier De La Rubia, Mercedes Gironella, Luis Palomera, Albert Oriol, Maria Casanova, Valentin Cabañas, Francisco Taboada, Albert Pérez-Montaña, Felipe De Arriba, Noemi Puig, Gonzalo Carreño-Tarragona, Santiago Barrio, Jose Enrique de la Puerta, Angel Ramirez-Payer, Isabel Krsnik, Juan Jose Bargay, Juan Jose Lahuerta, Maria-Victoria Mateos, Jesus F. San-Miguel, Bruno Paiva, Joaquin Martinez-Lopez

**Affiliations:** 1 Hospital 12 de Octubre, Madrid, CNIO, Universidad Complutese, Madrid, Spain; 2 Clinica Universidad de Navarra, Centro de Investigacion Medica Aplicada (CIMA), IDISNA, CIBERONC Pamplona, Pamplona, Spain; 3 Clinical and Traslational Lung Cancer Research Unit, i+12 Research Institute and Biomedical Research Networking Center in Oncology (CIBERONC), Madrid, Spain; 4 Universidad de Cantabria, Hospital Universitario Marqués de Valdecilla, Santander, Spain; 5 Hospital de Cabueñes, Gijon, Spain; 6 Hospital Clínico Universitario de Valladolid, Valladolid, Spain; 7 Hospital Doctor Peset, Valencia, Spain; 8 Hospital Universitari Vall d’Hebron, Barcelona, Spain; 9 Hospital Clinico Universitario Lozano Blesa, Zaragoza, Spain; 10 Hospital German Trias i Pujol, Badalona, Spain; 11 Hospital Costa del Sol, Marbella, Spain; 12 Hospital Clínico Universitario Virgen de la Arrixaca, Murcia, Spain; 13 Hospital Universitario Central de Asturias (HUCA), Oviedo, Spain; 14 Hospital Son Espases, Palma, Spain; 15 Hospital Universitario Morales Meseguer. IMIB-Arrixaca, Murcia, Spain; 16 Hospital Universitario de Salamanca, Instituto de Investigacion Biomedica de Salamanca (IBSAL), Centro de Investigación del Cancer (IBMCC-USAL, CSIC), Salamanca, Spain; 17 Hospital de Galdakao, Vizcaya, Spain; 18 Hospital Puerta de Hierro, Madrid, Spain; 19 Hospital Universitario Son Llàtzer, Palma, Spain

**Keywords:** Cancer genomics, Translational research

## To the Editor:

Sequence-based analysis has come to play an integral role in many hematological malignancies [1], but disorders such as systemic light-chain (AL) amyloidosis remain poorly characterized due to its low incidence and small tumor size [2, 3]. Thus, greater knowledge about the immunogenetic landscape of AL amyloidosis is required since, for example, potential differences between the genomic profiles of AL amyloidosis and multiple myeloma (MM) could help identifying patients with monoclonal gammopathies at greater risk of developing AL amyloidosis and monitor presymptomatic organ damage [4, 5].

To gain further insight into the immunogenetic landscape of AL amyloidosis, we performed whole-exome sequencing (WES) on highly purified bone marrow clonal plasma cells (PCs) isolated by fluorescence activation cell sorting (FACS) based on patient-specific aberrant phenotypes. A total of 27 patients with confirmed new diagnosis of AL amyloidosis based on the presence of amyloid-related systemic syndrome, positive amyloid tissue staining with Congo red, restricted light-chain deposition by immunohistochemistry or mass spectometry, and evidence of PC clonality were investigated. Patients’ demographics and clinical characteristics are described in Supplementary Table 1. PCs were collected and processed in triplicates followed by whole genome amplification of samples with genomic DNA amounts <50 ng (Supplementary Table 2). Afterwards, library construction, exome enrichment, and sequencing were performed individually. An overall average depth of 63× and mean on-target coverage of 84% were obtained. Data were deposited in the Sequence Read Archive of the NCBI (http://www.ncbi.nlm.nih.gov/sra) under the PRJNA596656 access number. To increase specificity, only single-nucleotide variants (SNVs) and indels detected by both Strelka [6] and Varscan2 [7] variant callers were selected. Moreover, only somatic mutations present in two of three libraries per patient were considered positive. Germline variants were excluded through WES of matched peripheral blood cells. The mutational profile of patients with AL amyloidosis was compared with that of patients with MM enrolled in CoMMpass (*n* = 930; IA13c dataset). The CNVKit [8] was used to determine copy number abnormalities (CNA) from WES data (in 21 of the 27 AL patients). Deep sequencing of B-cell receptor immunoglobulin (BcR Ig) gene rearrangements was performed in all patients as previously described [9], and compared with that of a series of 62 newly diagnosed MM patients. Detailed methodology is available in the Supplementary methods.

**Fig. 1:**
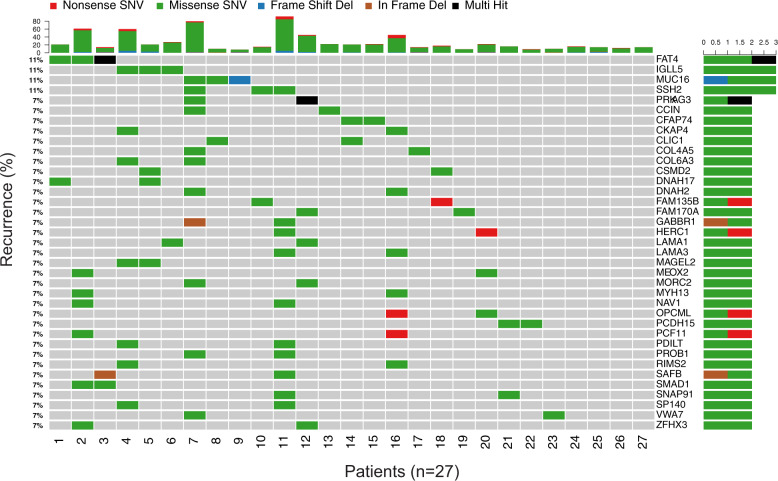
Genes recurrently mutated in AL. Distribution per patient of the most frequently mutated genes (*n* = 37). Boxes were colored according to the type of mutation. Top barplots define the total number of mutations per patient.

**Fig. 2:**
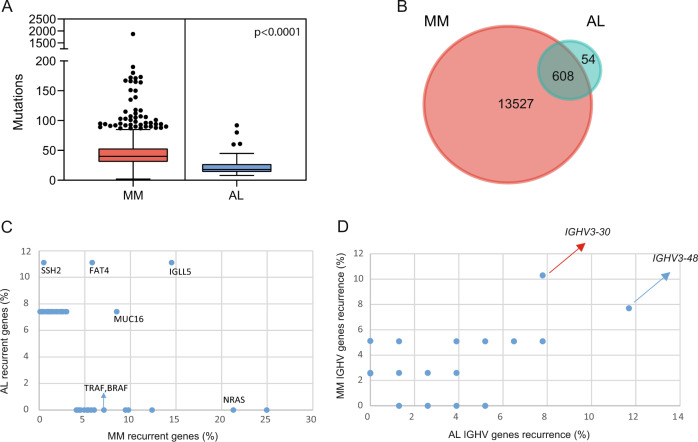
The mutational landscape in AL and MM. **a** Number of mutations in patients with newly diagnosed AL and MM. **b** Shared and private mutations between AL and MM. **c** Driver genes based in its recurrence in AL and MM. **d** Predominant Ig heavy chain gene rearrangements used in AL and MM.

We identified a total of 718 exonic, non-immunoglobulin, nonsynonymous mutations with a variant allelic fraction (VAF) >5% (683 SNV and 35 indel). Total number and type of mutations are described in Supplementary Fig. 1. Interestingly, mutational burden was significantly correlated with patients’ age (*R* = 0.51, *p* < 0.001) (Supplementary Fig. 2) though not tumor burden (data not shown). Only 37 out of 662 (5.5%) mutated genes were altered more than once (Fig. 1). That notwithstanding, 23 of the 27 cases (85%) presented with at least one mutation in one of the 37 genes (range, 1–11); being *FAT4*, *IGLL5*, *MUC16*, and *SSH2* the most frequently mutated genes (≥3 patients). With a median of 18 mutations per sample (range, 8–92), patients with AL amyloidosis are closer to monoclonal gammopathy of undetermined significance (MGUS) (median of 19) [10] rather than MM (median of 38 in the CoMMpass IA13c dataset, *p* < 0.0001; Fig. 2a) in terms of mutational load. By contrast, the presence of CNA was more frequent in AL amyloidosis (19/21, 90.5%; Supplementary Fig. 3) than MGUS (60.6% in Mikulasova et al.) [10] and similar to MM patients (virtually 100%) [11]. Overall, these results underpin recent observations based on the immunophenotypic characterization of clonal PCs [5], and locate AL amyloidosis in the crossroad between MGUS and MM also in genetic grounds. Of note, the only alterations associated with inferior progression-free survival were gains in chromosomes 9 and 19 (Supplementary Fig. 4a, b), whereas del(13q) was associated with higher NT-proBNP levels (Supplementary Fig. 5). Furthermore, patients with +1q also displayed greater risk of cardiac involvement (Supplementary Table 3).

Interestingly, various MM-defined driver mutations [12] were undetected in patients with AL amyloidosis (e.g., *NRAS*, *BRAF*, and *TRAF*) or observed only once (e.g., *DIS3* and *DUSP2*), most of them being subclonal with a median VAF of 23% (Supplementary Fig. 6) that was unrelated to the lower tumor burden in AL amyloidosis due to the FACSorting strategy used to isolate clonal PCs, as described above. Simultaneous analysis of ours and the CoMMpass datasets unveiled that out of 14,135 mutated genes, only 608 (4.4%) were shared between AL amyloidosis and MM (Fig. 2b), with considerable differences in their recurrence (Fig. 2c). Of note, none of the 65 genes exclusively mutated in AL amyloidosis were recurrent. Furthermore, only four genes (*XKR5*, *PRSS45, PKD1L2, and SRRM5*) overlapped with the 105 described by Boyle et al. [3] as AL restricted. While the results from these two studies suggest that MM recurrent mutations are unfrequently detected in patients with AL amyloidosis, recent data based on WES and targeted sequencing reported by Huang et al. [13] identified recurrent mutations in *KRAS*. Furthermore, *IGLL5* emerged as one of the most commonly mutated genes in ours and the latter series, but not in that analyzed by Boyle et al. We have found no association between mutated genes and patients’ outcome, whereas Huang et al. identified three mutated genes with prognostic value. Altogether, further studies in larger series of patients are warranted to shed more light into the mutational landscape and potential clinical correlations in AL amyloidosis.

In the absence of a unifying genetic event defining AL amyloidosis, BcR Ig gene rearrangements emerge as an alternative to understand the propensity for the deposition of monoclonal Ig light-chains in the form of β-sheet fibrils, as well as organ tropism. Previous studies based on PCR amplification of individual (typically Lambda) light-chain variable gene (VL) families have shown a potential bias in germline donor use in patients with AL amyloidosis [14] and that VL gene usage may influence clinical presentation, organ deposition, and outcome [15, 16]. Hence, we performed next-generation sequencing (NGS) of Ig heavy chain gene (IGH) and Kappa light-chain (IGK) rearrangements both in patients with AL amyloidosis (*n* = 27) and MM (*n* = 63) to provide complementary information on IGH and IGK repertoires, clonal variability, and extent of somatic hypermutations. A total of 39 IGHV-D-J and IGK-V-J clonotypes were identified and 5 of the 27 (19%) patients with AL amyloidosis displayed more than two clonal rearrangements. This extent of clonal heterogeneity differs (*p* = 0.024) from that found in our MM series (3.9%). Using a cut-off of 98% to define homology, 9 of 39 sequences (23%) in AL amyloidosis were considered as mutated, which is slightly less when compared with that found in MM (36%). The CDR3 length was not significantly different between AL amyloidosis and MM (median of 54 versus 51 amino acids). The most frequent IGH gene involved in AL amyloidosis was *IGHV3-48* (recurrence of 10.3%) and 100% of patients who have this rearrangement had kidney involvement (*p* = 0.025) (Supplementary Table 4), whereas *IGHV3-30* was the most recurrent (12%) in MM (Fig. 2d). Thus, expression of *IGHV3-48* adds up to *IGLV6-57* as germline Ig genes associated with dominant kidney deposition [14, 15]. Of note, we found no significant differences regarding IGH and IGK repertoires, clonal variability, and extent of somatic hypermutations between patients with light-chain only versus heavy- and light-chain M-component (data not shown).

This study confirms previous observations that AL amyloidosis cannot be defined by a singular or a set of well-defined genetic events. In fact, based on combined results from WES of 99 patients (27 in this study, 24 in Boyle et al. [3], and 48 in Huang et al. [13]), 63 genes were found to be recurrently mutated. By contrast, our study further supports the notion that germline Ig gene use is a key determinant in the pathogenesis of AL amyloidosis [16], and unveils novel associations of cytogenetic abnormalities with organ involvement and outcome. The extent of CNA was similar between AL and MM, but MM-driver genes were not recurrently mutated in AL, which instead was marked by greater clonal heterogeneity (i.e., similarly to MGUS) [10]. Taken together with differential predominance of IGH rearrangements, our results suggest that amongst other factors, Ig germline genes rather than specific cytogenetic abnormalities predispose light-chains from a PC clone to adopt an aberrant conformation, typically closer to patients’ MGUS stage before the development of higher tumor burden and accumulation of MM-driver mutations. Given the high frequency of Lambda light-chain deposition in AL, further studies are warranted to investigate if clonal mutations in *IGLL5* (median VAF, 64%) contribute to this process. In such cases, its detection together with other candidate genes (e.g., *FAT4*, *MUC16*, and *SSH2*) through NGS diagnostics could emerge as novel risk markers for AL amyloidosis in patients with monoclonal gammopathies.

## Supplementary information

Supplemental table 1

Supplemental table 2

Supplemental table 3

Supplemental table 4

Supplemental material

Supplemental figure 1

Supplemental figure 2

Supplemental figure 3

Supplemental figure 4

Supplemental figure 5

Supplemental figure 6
