# Supplementary material for: Immunogenetic characterization of clonal plasma cells in systemic light-chain amyloidosis
Source: Leukemia. 2020 Mar 19;35(1):245–9. doi: 10.1038/s41375-020-0800-6 (PMC7787969; doi:10.1038/s41375-020-0800-6)
Supplement: Supplementary file 1 — Supplemental table 1 [file 41375_2020_800_MOESM1_ESM.docx]

**Supplemental Table 1.** Patient demographics and clinical characteristics of immunoglobulin light-chain amyloidosis (AL) patients (N=27).

**Patient demographics and clinical characteristics Distribution**

| Male / Female* | 70% / 30% |
| --- | --- |
| Age (years)¥ | 67 (45-78) |
| Isotype* |  |
| Free Kappa | 19% |
| Free Lambda | 26% |
| IgG-Kappa | 11% |
| IgG-Lambda | 37% |
| IgA-Lambda | 7% |
| No. of organs involved* |  |
| Kidney involvement* | 48% |
| Heart involvement* | 52% |
| Liver involvement* | 4% |
| Peripheral neuropathy* | 7% |
| Gastrointestinal involvement* | 22% |
| Revised Mayo Clinic stage* |  |
| I | 5% |
| II | 50% |
| III | 30% |
| IV | 15% |
| Serum albumin (g/dL)¥ | 3.6 (1.6-5) |
| Serum creatinine (mg/dL)¥ | 1 (0.7-7) |
| β2-microglobulin (mg/L)¥ | 2.8 (0.2-18) |
| Serum M-component (g/dL)¥ | 0.62 (0-7.4) |
| Urine M-component (g/24h)¥ | (0-3.7) |
| dFLC (mg/L)¥ | 227 (2.5-18899) |
| Serum cardiac troponin T (ng/mL)¥ | 0.08 (0.005-51) |
| NT-ProBNP (pg/mL)¥ | 765 (12-26680) |
| % plasma cells by morphology¥ | 10 (2-23) |
| % plasma cells by MFC¥ | 0.1 (0.02-3.6) |
| Therapy |  |
| High-dose | 7% |
| Mel/CP-Dex | 22% |
| Bortezomib + CP/Mel | 52% |
| Others | 19% |

|  |  |
| --- | --- |

Results expressed as median (range) ¥ or as percentage of cases*. PC: plasma cells; NT-ProBNP: N-terminal prohormone of brain natriuretic peptid; dFLC difference between the involved and uninvolved serum free-light chains; Mel: melphalan; Dex: dexamethasone; CP: cyclophosphamide.
