# Supplementary material for: Immunogenetic characterization of clonal plasma cells in systemic light-chain amyloidosis
Source: Leukemia. 2020 Mar 19;35(1):245–9. doi: 10.1038/s41375-020-0800-6 (PMC7787969; doi:10.1038/s41375-020-0800-6)
Supplement: Supplementary file 2 — Supplemental table 2 [file 41375_2020_800_MOESM2_ESM.docx]

**Supplemental Table 2.** DNA concentration and genomic amplification of samples. repli-G amplified samples are also specified.

| **Patients** | **Samples** | **Total ng** | **repli-G** |
| --- | --- | --- | --- |
| Patient 1 | S_1_1 | 742 | No |
|  | S_1_2 | 593.6 |  |
|  | S_1_3 | 445.2 |  |
| Patient 2 | S_2_1 | 1156 | No |
|  | S_2_2 | 1040.4 |  |
|  | S_2_3 | 924.8 |  |
| Patient 3 | S_3_1 | < 50 ng | Yes |
|  | S_3_2 |  |  |
|  | S_3_3 |  |  |
| Patient 4 | S_4_1 | 1240 | No |
|  | S_4_2 | 1116 |  |
|  | S_4_3 | 992 |  |
| Patient 5 | S_5_1 | < 50 ng | Yes |
|  | S_5_2 |  |  |
|  | S_5_3 |  |  |
| Patient 6 | S_6_1 | 1324 | No |
|  | S_6_2 | 1059.2 |  |
|  | S_6_3 | 794.4 |  |
| Patient 7 | S_7_1 | < 50 ng | Yes |
|  | S_7_2 |  |  |
|  | S_7_3 |  |  |
| Patient 8 | S_8_1 | 428 | No |
|  | S_8_2 | 342 |  |
|  | S_8_3 | < 50 ng | Yes |
| Patient 9 | S_9_1 | < 50 ng | Yes |
|  | S_9_2 |  |  |
|  | S_9_3 |  |  |
| Patient 10 | S_10_1 | 399.6 | No |
|  | S_10_2 | 239.8 |  |
|  | S_10_3 | < 50 ng | Yes |
| Patient 11 | S_11_1 | < 50 ng | Yes |
|  | S_11_2 |  |  |
|  | S_11_3 |  |  |
| Patient 12 | S_12_1 | 432 | No |
|  | S_12_2 | 259.2 |  |
|  | S_12_3 | < 50 ng | Yes |
| Patient 13 | S_13_1 | 369.2 | No |
|  | S_13_2 | 276.9 |  |
|  | S_13_3 | < 50 ng |  |
| Patient 14 | S_14_1 | 668 | No |
|  | S_14_2 | 601.1 |  |
|  | S_14_14 | 534.4 |  |
| Patient 15 | S_15_1 | 2900 | No |
|  | S_15_2 | 2320 |  |
|  | S_15_3 | 1740 |  |
| Patient 16 | S_16_1 | < 50 ng | Yes |
|  | S_16_2 |  |  |
|  | S_16_3 |  |  |
| Patient 17 | S_20_1 | < 50 ng | Yes |
|  | S_20_2 |  |  |
|  | S_20_3 |  |  |
| Patient 18 | S_18_1 | 404 | No |
|  | S_18_2 | 303 |  |
|  | S_18_3 | < 50 ng |  |
| Patient 19 | S_19_1 | < 50 ng | Yes |
|  | S_19_2 |  |  |
|  | S_19_3 |  |  |
| Patient 20 | S_20_1 | 1200 | No |
|  | S_20_2 | 1080 |  |
|  | S_20_3 | 960 |  |
| Patient 21 | S_21_1 | 412 | No |
|  | S_21_2 | 247.2 |  |
|  | S_21_3 | < 50 ng | Yes |
| Patient 22 | S_22_1 | 1412 | No |
|  | S_22_2 | 1129.6 |  |
|  | S_22_3 | 847.2 |  |
| Patient 23 | S_23_1 | 428 | No |
|  | S_23_2 | 256.8 |  |
|  | S_23_3 | < 50 ng | Yes |
| Patient 24 | S_24_1 | 424 | No |
|  | S_24_2 | 318 |  |
|  | S_24_3 | < 50 ng |  |
| Patient 25 | S_25_1 | 660 | No |
|  | S_25_2 | 528 |  |
|  | S_25_3 | 396 |  |
| Patient 26 | S_26_1 | < 50 ng | Yes |
|  | S_26_2 |  |  |
|  | S_26_3 |  |  |
| Patient 27 | S_27_1 | 564 | No |
|  | S_27_2 | 507.6 |  |
|  | S_27_3 | 451.2 |  |
