# Supplementary material for: Immunogenetic characterization of clonal plasma cells in systemic light-chain amyloidosis
Source: Leukemia. 2020 Mar 19;35(1):245–9. doi: 10.1038/s41375-020-0800-6 (PMC7787969; doi:10.1038/s41375-020-0800-6)
Supplement: Supplementary file 3 — Supplemental table 3 [file 41375_2020_800_MOESM3_ESM.docx]

**Supplemental Table 3**. Contingency table regarding the presence of 1q+ and cardiac involvement.

|  | | | | |  |
| --- | --- | --- | --- | --- | --- |
|  |  |  |  |  |  |
|  | | **1q+** | | Total |  |
|  |  | - | + |  |  |
| **Cardiac involvement** | - | 10 (67%) | 1 (17%) | 11 |  |
|  | + | 5 (33%) | 5 (83%) | 10 |  |
| Total | | 15 | 6 | 21 |  |

## Pearson's χ2= 4.295. Significance= 0.038. Fisher’s Exact test= 0.063
