# Supplementary material for: Immunogenetic characterization of clonal plasma cells in systemic light-chain amyloidosis
Source: Leukemia. 2020 Mar 19;35(1):245–9. doi: 10.1038/s41375-020-0800-6 (PMC7787969; doi:10.1038/s41375-020-0800-6)
Supplement: Supplementary file 4 — Supplemental table 4 [file 41375_2020_800_MOESM4_ESM.docx]

**Supplemental Table 4**. Contingency table of patients with IGHV3-48 rearrangement and kidney involvement.

|  | | | | |  |
| --- | --- | --- | --- | --- | --- |
|  |  |  |  |  |  |
|  | | **Presence of IGHV3-48 rearrangement** | | Total |  |
|  |  | - | + |  |  |
| **Kidney involvement** | - | 14 (61%) | 0 (0%) | 14 |  |
|  | + | 9 (39%) | 4 (100%) | 13 |  |
| Total | | 23 | 4 | 27 |  |

## Pearson's χ2= 5.057. Significance= 0.025. Fisher’s Exact Test= 0.041
