# Supplementary material for: Immunogenetic characterization of clonal plasma cells in systemic light-chain amyloidosis
Source: Leukemia. 2020 Mar 19;35(1):245–9. doi: 10.1038/s41375-020-0800-6 (PMC7787969; doi:10.1038/s41375-020-0800-6)
Supplement: Supplementary file 5 — Supplemental material [file 41375_2020_800_MOESM5_ESM.docx]

**Immunogenetic characterization of clonal plasma cells in systemic light-chain amyloidosis**

**Supplemental material**

**Supplemental Tables and Figures:** 4+6

**Supplemental index:**

**Supplemental Methods**

**Supplemental Table 1.** Patient demographics and clinical characteristics of immunoglobulin light-chain amyloidosis (AL) patients (N=27).

**Supplemental Table 2**. Summary of total genomic DNA isolated per sample. repli-G amplified samples are also specified.

**Supplemental Table 3**. Contingency table regarding the presence of 1q+ and cardiac involvement.

**Supplemental Table 4**. Contingency table of patients with IGHV3-48 rearrangement and kidney involvement.

**Supplemental Figure 1**. Summary of genomic distribution and functional consequences of SNV (A) and INDEL (B) alterations in AL series.

**Supplemental Figure 2**. Correlation analysis between age and tumor burden.

**Supplemental Figure 3.** Frequency plot providing an overview of the distributions of gains (red) and losses (blue) as percentages for all 21 patients.

**Supplemental Figure 4**. Progression free survival (PFS) of AL patients. A) Gains in chromosomes 9 vs other patients, B) Gains in chr 19 vs other patients.

Chr: Chromosome

**Supplemental Figure 5.** Box plot showing the association of 1 del(13q) was associated with higher NT-proBNP levels**.** Box plot rectangles show the interquartile ranges (IQR) from the first quartile to the third quartile and the lines in the middle of the boxes represent the medians. The whiskers are drawn to the nearest value not exceeding 1.5 times the IQR.

**Supplemental Figure 6.** VAF distribution of the 63 MM-driver mutations described by Walker et al.^1^ in the CoMMpass IA13c dataset vs our AL series. Box plot rectangles show the interquartile ranges (IQR) from the first quartile to the third quartile and the lines in the middle of the boxes represent the medians. The whiskers are drawn to the nearest value not exceeding 1.5 times the IQR.

**Supplemental Methods**

**Patients**

We analyzed 27 light-chain amyloidosis (AL) patients with confirmed diagnosis of AL based on the presence of amyloid-related systemic syndrome, positive amyloid tissue staining with Congo red, and evidence of PC clonality. Organ involvement was defined following the recommendations of Mayo Stratification of Myeloma and Risk-Adapted Therapy (mSMART) Consensus Statement.^2^

Samples were collected through a multicenter study (Hospital Universitario de Salamanca, Clínica Universidad de Pamplona, and Hospital 12 de Octubre), and with the support of the Spanish myeloma group (GEM) after informed consent was given, in accordance with the local ethics committee guidelines and the Helsinki Declaration.

**Samples and CD138 isolation**

BM aspirates were immunophenotyped using 8-color combinations of monoclonal antibodies, and a direct immunofluorescence stain-and-then-lyse technique (BV421HV450/ BV510/ FITC/ PE/ PerCP-Cy5.5/ PE-Cy7/ APC/ APC-H7): CD138/ CD27/ CD38/ CD56/ CD45/ CD19/ CD117/ CD81. Data acquisition was performed with a FACSCantoII flow cytometer (Becton Dickinson Biosciences – BD, San Jose, CA) using the FACSDiva 6.1 software (BD), and following the EuroFlow guidelines^3^. Expression profiles (iPEP) based on the 28 (8+20) different markers evaluated at the single cell level was performed with the Infinicyt software (Cytognos SL, Salamanca, Spain). Clonal plasma cells were sorted (FACSAria II, Becton Dickinson Biosciences – BD, San Jose, CA; purity ≥97%) according to patient-specific aberrant phenotypes.

**DNA isolation**

We analyzed a total of 54 paired samples, 27 samples of sorted clonal plasma cells, and 27 samples of peripheral blood from the same patient as a control to discard germline variants. Genomic DNA was extracted using QIAamp DNA Mini and Micro Kits (QIAGEN) for peripheral blood and sorted plasma cells respectively.

**Library preparation and sequencing**

Whole-exome (WES) capture libraries were generated with the SureSelectQ^XT^ Human All Exon V6 + UTRs Reagent Kit (Agilent Technologies) for Illumina sequencing from 50ng of genomic DNA both tumor and control samples, following manufacturer instructions. Those samples with less than 50ng were amplified by repli-G, as previouslly described^4^.

For consistency purpose we generated three independent libraries from tumor samples in order discard sequencing artifacts in the bioinformatic analysis. Libraries were multiplexed and 2x150bp paired-end sequencing was performed on an Illumina NextSeq 500 platform (High Output Kit v2).

**Bioinformatic analysis**

**Variant analysis workflow**

Sequencing reads quality were evaluated using the FastQC tool^5^. Each replicate was analyzed independently. FASTQ files of all samples were aligned to hg19 human reference with the BWA mem (Burrows–Wheeler aligner-maximum exact matches) aligner, and SNV and small INDELs were analyzed with two different methods Strelka^6^ and Varscan2^7^ using default options.

MAFtools^8^ (https://github.com/PoisonAlien/maftools/) was used for SNV analysis and visualization. Mean depth coverage of 63x was achieved, and the mean on-target coverage was of 84% with 87% (median) of target regions covered by at least 50x. For functional annotation we used the software ANNOVAR.^9^ Then, in-house script was used for variant filtering and somatic variant candidate selection. SNVs and small INDELs were considered if they were present in at least two replicates, their VAF was ≥5% and they had a minor allele frequency (MAF) <1% in any population from Exome Aggregation Consortium (ExAC) database. A total of 718 mutations were identified and manually reviewed with the Integrative Genomics Viewer (IGV) visualization tool^10^ to discard sequencing artifacts. CNVKit software was employed for CNV identification which achieved higher resolution in copy number changes from WES data^11^ by using targeted reads and the off-target reads. Accurate identification of CNV was not possible in 6 samples with repli-G amplification performed in all 3 replicates.

**Immunoglobulin repertoire analysis**

The analysis of IGHV-D-J and IGKV-J genes was realized with the same samples used for WES analysis. The amplification of IGH/IGK rearrangements at DNA level was performed according to the previously published^12^. The libraries were generated by ligation of specific adaptor oligos (NEBNext® Fast DNA Library Prep Set for Ion Torrent™kit) and sequenced on an Ion S5 sequencer (ThermoFisher Scientifc, Palo Alto, CA, USA).

The sequencing data were analyzed with MiXCR, a universal framework that processes big immunome data from raw sequences to identify and count clonotypes. MiXCR efficiently handles paired- and single-end reads, considers sequence quality, corrects PCR errors and identifies germline hypermutations.^13^ We analyzed the sequences aligning to the IMGT database (http://www.imgt.org). A clonotype was identified when at least the unique rearrangements was present at a frequency above 10%. Sequences with a germline homology of 98% or higher in IGHV genes were considered as unmutated, and those with a homology less than 98% were considered as mutated.

**Multiple myeloma dataset**

Somatic SNV from CoMMpass IA13c dataset was downloaded from MMRF project (www.themmrf.org) (930 patients). Dataset was fully re-annotated by ANNOVAR.^9^ To make our data as comparable as possible, we use the same filtering criteria for AL. Of the total number of patients, only the samples at diagnosis and from bone marrow were selected. Then, we kept only mutations obtained by Strelka and discard variants with VAF≤5%, MAF>1%, UTR and synonymous, IG genes, intronic and non-coding mutations.

**Statistical analysis**

Statistical analysis was performed using SPSS v22 and GraphPad Prism v8 softwares. We performed t-test on parametric variables, and Mann-Whitney on non-parametric variables. For progression-free survival analysis (PFS) we used Kaplan-Meier curves and two-sided long-rank test. Data were considered statistically significant when p<0.05.

**Supplemental references**

1 Walker BA, Mavrommatis K, Wardell CP, Ashby TC, Bauer M, Davies FE *et al.* Identification of novel mutational drivers reveals oncogene dependencies in multiple myeloma. *Blood* 2018; **132**: 587–597.

2 Dispenzieri A, Buadi F, Kumar SK, Reeder CB, Sher T, Lacy MQ *et al.* Treatment of Immunoglobulin Light Chain Amyloidosis: Mayo Stratification of Myeloma and Risk-Adapted Therapy (mSMART) Consensus Statement. *Mayo Clin Proc* 2015; **90**: 1054–1081.

3 Kalina T, Flores-Montero J, van der Velden VHJ, Martin-Ayuso M, Böttcher S, Ritgen M *et al.* EuroFlow standardization of flow cytometer instrument settings and immunophenotyping protocols. *Leukemia* 2012; **26**: 1986–2010.

4 Pinard R, de Winter A, Sarkis GJ, Gerstein MB, Tartaro KR, Plant RN *et al.* Assessment of whole genome amplification-induced bias through high-throughput, massively parallel whole genome sequencing. *BMC Genomics* 2006; **7**: 216.

5 *Babraham Bioinformatics - FastQC A Quality Control tool for High Throughput Sequence Data*. http://www.bioinformatics.babraham.ac.uk/projects/fastqc/ (accessed 24 Jan2019).

6 Saunders CT, Wong WSW, Swamy S, Becq J, Murray LJ, Cheetham RK. Strelka: accurate somatic small-variant calling from sequenced tumor–normal sample pairs. *Bioinformatics* 2012; **28**: 1811–1817.

7 Koboldt DC, Chen K, Wylie T, Larson DE, McLellan MD, Mardis ER *et al.* VarScan: variant detection in massively parallel sequencing of individual and pooled samples. *Bioinforma Oxf Engl* 2009; **25**: 2283–2285.

8 Mayakonda A, Lin D-C, Assenov Y, Plass C, Koeffler HP. Maftools: efficient and comprehensive analysis of somatic variants in cancer. *Genome Res* 2018; **28**: 1747–1756.

9 Wang K, Li M, Hakonarson H. ANNOVAR: functional annotation of genetic variants from high-throughput sequencing data. *Nucleic Acids Res* 2010; **38**: e164.

10 Robinson JT, Thorvaldsdóttir H, Winckler W, Guttman M, Lander ES, Getz G *et al.* Integrative Genomics Viewer. *Nat Biotechnol* 2011; **29**: 24–26.

11 Talevich E, Shain AH, Botton T, Bastian BC. CNVkit: Genome-Wide Copy Number Detection and Visualization from Targeted DNA Sequencing. *PLOS Comput Biol* 2016; **12**: e1004873.

12 Martinez-Lopez J, Sanchez-Vega B, Barrio S, Cuenca I, Ruiz-Heredia Y, Alonso R *et al.* Analytical and clinical validation of a novel in-house deep-sequencing method for minimal residual disease monitoring in a phase II trial for multiple myeloma. *Leukemia* 2017; **31**: 1446–1449.

13 Bolotin DA, Poslavsky S, Mitrophanov I, Shugay M, Mamedov IZ, Putintseva EV *et al.* MiXCR: software for comprehensive adaptive immunity profiling. *Nat Methods* 2015; **12**: 380–381.
