# Supplementary material for: Immunogenetic characterization of clonal plasma cells in systemic light-chain amyloidosis
Source: Leukemia. 2020 Mar 19;35(1):245–9. doi: 10.1038/s41375-020-0800-6 (PMC7787969; doi:10.1038/s41375-020-0800-6)

Supplemental Figure 1. Summary of genomic distribution and functional consequences of SNV (A) and INDEL (B) alterations in AL series.

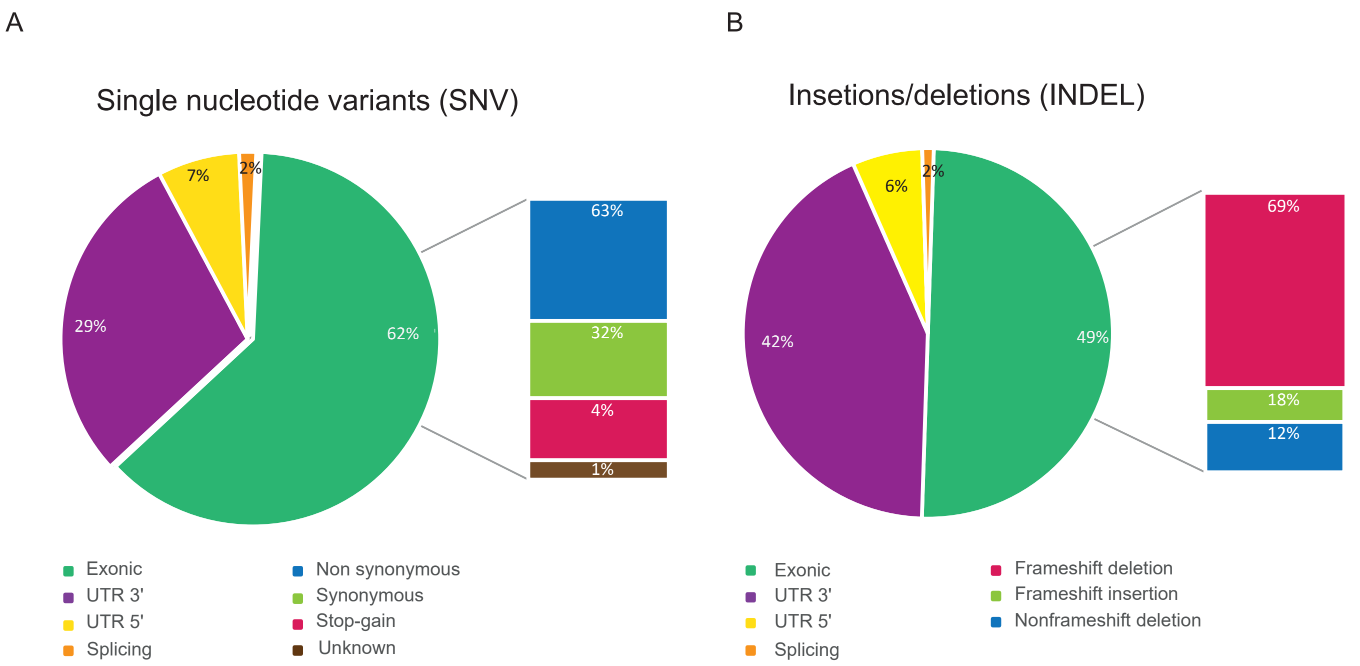

Supplement: Supplementary file 6 — Supplemental figure 1 [file 41375_2020_800_MOESM6_ESM.pdf]
