# Supplementary figures and images for: Immunogenetic characterization of clonal plasma cells in systemic light-chain amyloidosis
Source: Leukemia. 2020 Mar 19;35(1):245–9. doi: 10.1038/s41375-020-0800-6 (PMC7787969; doi:10.1038/s41375-020-0800-6)

**Supplemental Figure 2.** Correlation analysis between age and tumor burden.

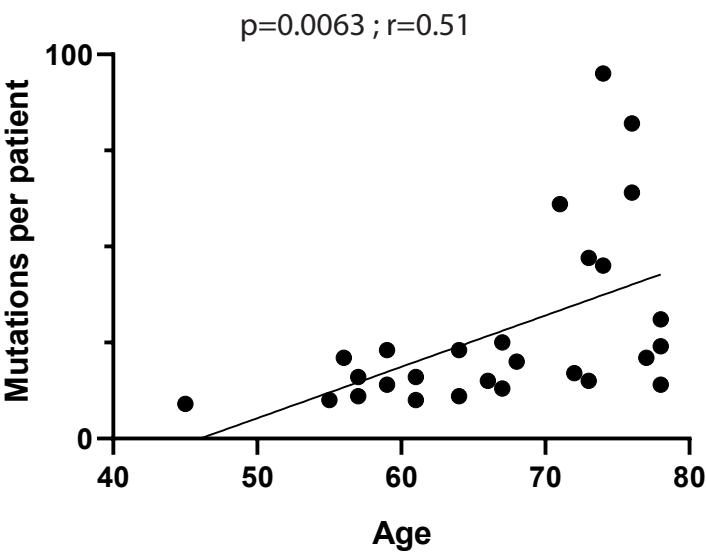

Supplement: Supplementary file 7 — Supplemental figure 2 [file 41375_2020_800_MOESM7_ESM.pdf]
