# Supplementary material for: Immunogenetic characterization of clonal plasma cells in systemic light-chain amyloidosis
Source: Leukemia. 2020 Mar 19;35(1):245–9. doi: 10.1038/s41375-020-0800-6 (PMC7787969; doi:10.1038/s41375-020-0800-6)

Supplemental Figure 3. Frequency plot providing an overview of the distributions of gains (red) and losses (blue) as percentages for all 21 patients.

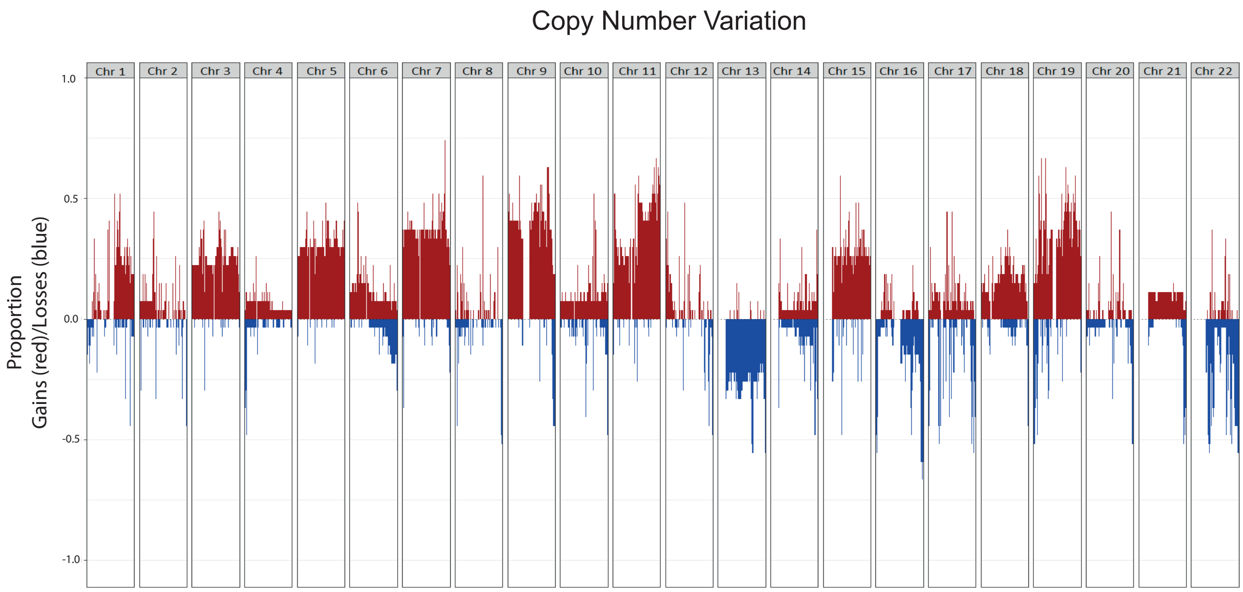

Supplement: Supplementary file 8 — Supplemental figure 3 [file 41375_2020_800_MOESM8_ESM.pdf]
