# Supplementary material for: Immunogenetic characterization of clonal plasma cells in systemic light-chain amyloidosis
Source: Leukemia. 2020 Mar 19;35(1):245–9. doi: 10.1038/s41375-020-0800-6 (PMC7787969; doi:10.1038/s41375-020-0800-6)

Supplemental Figure 4. Progression free survival (PFS) of AL patients. A) Gains in chromosomes 9 vs other patients, B) Gains in chr 19 vs other patients. Chr:Chromosome

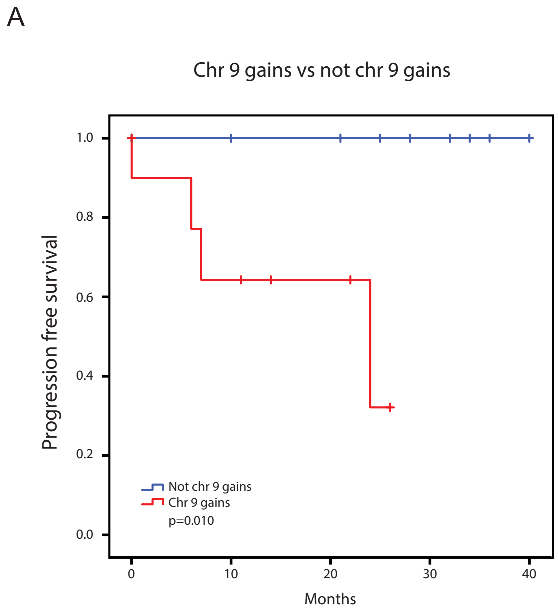

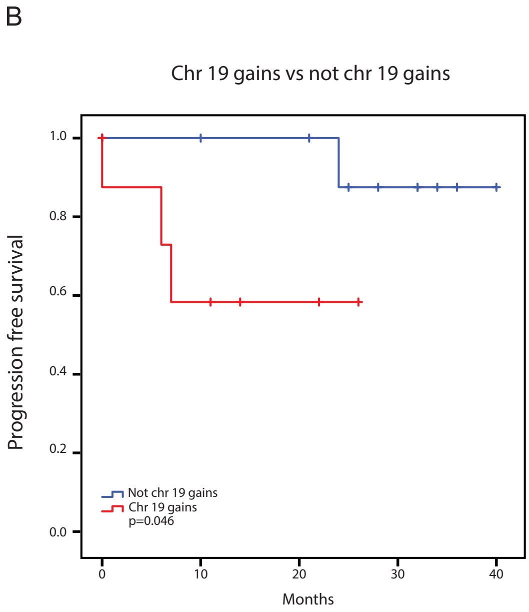

Supplement: Supplementary file 9 — Supplemental figure 4 [file 41375_2020_800_MOESM9_ESM.pdf]
