# Supplementary material for: Immunogenetic characterization of clonal plasma cells in systemic light-chain amyloidosis
Source: Leukemia. 2020 Mar 19;35(1):245–9. doi: 10.1038/s41375-020-0800-6 (PMC7787969; doi:10.1038/s41375-020-0800-6)

Supplemental Figure 5. Box plot showing the association of 1 del(13q) was associated with higher NT-proBNP levels. Box plot rectangles show the interquartile ranges (IQR) from the first quartile to the third quartile and the lines in the middle of the boxes represent the medians. The whiskers are drawn to the nearest value not exceeding 1.5 times the IQR.

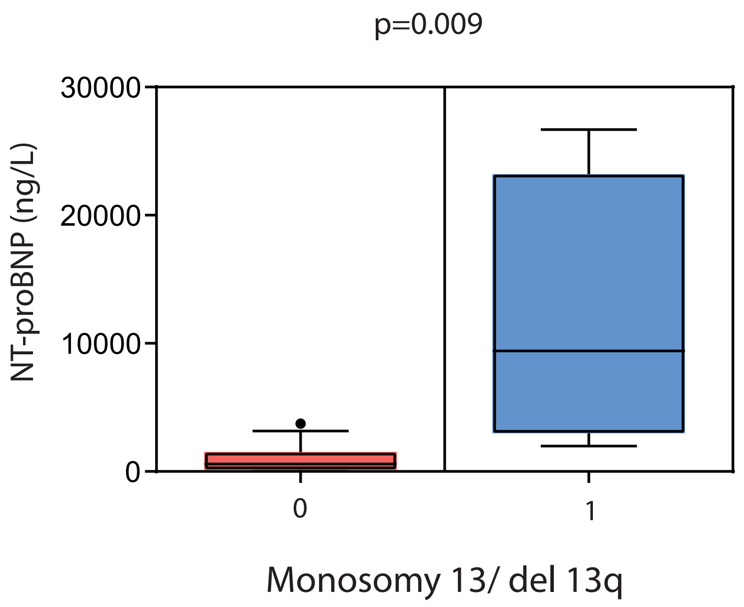

Supplement: Supplementary file 10 — Supplemental figure 5 [file 41375_2020_800_MOESM10_ESM.pdf]
